# Convalescent Plasma Coupled With Medications for the Treatment of a Severe COVID-19 Patient: Drugs Analysis and Pharmaceutical Care Based on the Newly Established Guidelines for COVID-19 Remedy

**DOI:** 10.3389/fphar.2020.00966

**Published:** 2020-06-24

**Authors:** Ying Wang, Yang Zhang, Qian Yu, Kun Zhu

**Affiliations:** ^1^ Department of Pharmacy, The Third Hospital of Jilin University, Jilin University, Changchun, China; ^2^ School of Biology and Food Engineering, Changshu Institute of Technology, Changshu, China

**Keywords:** convalescent plasma, COVID-19 (severe), drugs analysis, therapeutic strategy, guideline

## Abstract

Given the extreme importance of the current pandemic caused by COVID-19 and due to the fact that scientists agree that there is no identified treatment, this paper analyzes in detail the treatment of a severe COVID-19 patient with convalescent plasma and drugs based on current guidelines for COVID-19 diagnosis and treatment. This can provide a reference for other medical institutions on rational drug use and pharmaceutical care for severe COVID-19 patients.

## Introduction

In December 2019, several cases of viral pneumonia were reported in China. The whole-genome sequencing of the virus and the detection of pathogen nucleic acid revealed that the pathogen giving rise to this viral pneumonia with unknown cause was SARS-CoV-2 (Lai et al., 2020). The virus is highly infectious and causes lung infections that have been named Corona Virus Infective Disease-19 (COVID-19) by the World He5alth Organization. China has accumulated rich experience in the prevention and control of COVID-19. The National Health Commission (NHC) has issued several guidelines regarding the diagnosis and treatment of COVID-19 over time; these have recently been updated to the 7^th^ edition. COVID-19 patients are usually classified as having mild, common, severe, or critical illness (National Health Commission of the People's Republic of China, 2020), of which severe and critical COVID-19 patients possess poor prognosis and high mortality (Chen et al., 2020). At present, with regard to the remedy of severe and critical COVID-19 patients, there is still a lack of specific drugs, and experience of convalescent plasma (CP) therapy is also inadequate.

A patient with severe COVID-19 has been cured by a combination of CP and antiviral therapy in the Third Hospital of Jilin University (THJU). In the present work, the treatment plan and pharmaceutical care strategies of that severe patient are completely analyzed. It is hoped that the current work will give insight and a basis for rational drug use and pharmaceutical care of similar patients at other medical institutions.

## Therapeutic Process

On February 18, a 56-year-old male was diagnosed as “COVID-19” in the local primary hospital and was transferred to the THJU 2 days later. He was 172 cm tall and weighed 90 kg, with a corresponding body mass index of 30.4 kg/m^2^. On February 20, he was recorded to have temperature 36°C, pulse 82 times/min, breath rates 18 times/min, blood pressure 138/83 mmHg, and nucleic acid test (+). In chest CT, lamellar and flocculent ground-glass shadows could be seen below the pleura of both lungs. His full blood count results were white blood count 2.99×10^9^/L (4-10×10^9^/L), lymphocyte count 0.44×10^9^/L (0.8-4×10^9^/L), lymphocyte ratio 14.7% (20-50%), glutamic oxalacetic transaminase 84.0U/L (17-59U/L), γ-glutamyl transpeptidase 262.0U/L (15-73U/L), and hypersensitive c-reactive protein 63.6mg/L (0-3mg/L). Arterial blood gas analysis (nasal catheter for oxygen) showed PaCO_2_ 33 mmHg and PaO_2_ 54 mmHg. The diagnosis was COVID-19 (severe), Type I respiratory failure, Liver function abnormal.

After consultation with the COVID-19 Experts Group of Jilin Province, the patient was given noninvasive auxiliary ventilation (inspiratory pressure 12 cmH_2_O, expiratory pressure 6 cmH_2_O, oxygen concentration 65%), interferon (IFN), arbidol, lopinavir/ritonavir, glucocorticoid, and symptomatic treatment. α-2b IFN injection aerosol inhalation was administered at 5 million IU twice daily for 13 days (February 20–March 3), arbidol at 0.2 g tablet by mouth three times daily for 11 days (February 22–March 3), lopinavir/ritonavir (200 mg/50 mg) at 2 capsules by mouth twice daily for 11 days (February 22–March 3), and intravenous methylprednisolone sodium succinate at 40 mg twice daily for 3 days (February 21–23) then 20 mg twice daily for 1 day (February 24). On Day 2 of admission, fingertip oxygen saturation was 95%, and arterial blood gas analysis showed pH 7.50, PaCO_2_ 37 mmHg, PaO_2_ 74 mmHg, HCO_3_^-^ 28.9 mmol/L, Base Excess (BE) 5.5 mmol/L (noninvasive ventilator-assisted ventilation, inspiratory pressure of 12 cmH_2_O, expiratory pressure of 6 cmH_2_O, and oxygen concentration of 65%). According to the consultation and evaluation of the expert group, the patient met the requirements for CP therapy. On the second and fourth day of admission, 200 ml and 100 ml of CP were transfused, respectively. On the second day after the first transfusion of CP, the patient's symptoms were relieved. The parameters of noninvasive ventilation were adjusted to inspiratory pressure 12 cmH_2_O, expiratory pressure 6cmH_2_O, and oxygen concentration 50%, and fingertip oxygen saturation was 96%. On Day 6 of admission (the second day after the second CP transfusion), the parameters of the noninvasive ventilator were adjusted to an inspiratory pressure of 12cmH_2_O, expiratory pressure of 6cmH_2_O, and oxygen concentration of 45%, and fingertip blood oxygen saturation was 98%. On Day 10 of admission, the patient had good lung function. On Day 15 of admission, the noninvasive ventilator was stopped and was replaced by a nasal catheter (oxygen flow rate 4L/min). The oxygen saturation of fingertip blood was 97%. In addition, the leukocyte count, lymphocyte count, and lymphocyte ratio returned to normal on the second day after the first CP treatment. According to the evaluation of the COVID-19 Experts Group of Jilin Province, the patient met the discharge standard and was discharged on March 6. On March 20 and April 3, the patient was followed up and re-examined. He did not have COVID-19-related clinical symptoms, the SARS-CoV-2 nucleic acid tests (pharyngeal swab) were negative, and the laboratory and imaging results were normal.

## Treatment Analysis and Pharmaceutical Care

During the outbreak of the epidemic, people have been giving a great amount of attention to identifying effective drugs. Doctors and pharmacists should keep an eye on the updated guidelines. Comparisons and differences between the editions of these guidelines are summarized in **Table 1**.

**Table 1:** List of changes in diagnosis and treatment guidelines for COVID-19 (Wang et al., 2020).

Guidelines	Antimicrobial treatment	Antiviral therapy	Hormone therapy	CP therapy
**First edition** **(Tongji Hospital affiliated to Tongji Medical College, Huazhong University of Science and Technology edition)**	Avoid blind or inappropriate antimicrobial therapy, especially in combination with broad-spectrum antimicrobial agents. For patients with mild disease, administration of moxifloxacin or azithromycin orally or intravenously is recommended according to the patient's condition. For severe or critical patients, all possible pathogens should be empirically treated. For patients with sepsis, empirical antibiotics should be administered within 1 hour of the initial patient assessment.	There is currently no specific vaccine and no effective antiviral therapy (α-IFN injection can be tried, 5 million IU per time for adults, atomization inhalation bid; lopinavir/ritonavir, 2 pills per time, bid; if there is a history of endemic epidemiology or other infection-related risk factors, empirical therapeutic treatment can be provided, including oseltamivir or arbidol.	Routine use of corticosteroids should be avoided except for special reasons. Glucocorticoids can be used for 3-5 days according to the degree of respiratory distress and the progress of chest imaging. Dosage exceeding 1-2 mg·kg/·d equivalent of methylprednisolone is not recommended.	–
**Second edition**	Avoid blind or inappropriate antimicrobial therapy, especially in combination with broad-spectrum antimicrobial agents. Strengthen bacteriological surveillance and use antibiotics as soon as secondary infections occur.	Change: There is currently no specific vaccine and no effective antiviral therapy (α-IFN injection can be tried, 5 million IU per time for adults, atomization inhalation bid; lopinavir/ritonavir, 2 pills per time, bid.	Glucocorticoids can be used for 3-5 days according to the degree of respiratory distress and the progress of chest imaging. Dosage exceeding 1-2mg·kg/·d equivalent of methylprednisolone is not recommended.	–
**Third Edition**	Same as the second edition	Same as the second edition	Same as the second edition	–
**Fourth Edition**	Same as the second edition	Added: The recommended dosage of lopinavir/ritonavir is 400/100mg per time.	Same as the second edition	–
**Fifth Edition**	Avoid blind or inappropriate antimicrobial therapy, especially in combination with broad-spectrum antimicrobial agents.	Added: Intravenous ribavirin, 500mg/dose for adults, bid/tid. Note the adverse reactions and interactions associated with lopinavir/ritonavir, including diarrhea, nausea, vomiting, and liver impairment.	Added: High-dose glucocorticoid can inhibit the immune function and delay the clearance of coronavirus.	–
**Sixth Edition**	Same as the fifth edition	Added: Ribavirin: combined with IFN or lopinavir/ritonavir (400/100 mg for adults, bid, no more than 10 days). Chloroquine phosphate: 500 mg for adults, bid, no more than 10 days. Arbidol: 200 mg/dose for adults, tid, no more than 10 days. Stop the drug as soon as there is an intolerable side effect.	Same as the fifth edition	Yes
**Seventh Edition**	Same as the sixth edition	Chloroquine phosphate: adults 18–65 years old. Weight > 50 kg, 500 mg/dose, bid, 7 days; Weight < 50 kg, d1-d2, 500 mg/dose, bid, d3-d7, 500 mg/dose, qd. Chloroquine is forbidden in patients with heart disease. The number of weeks of pregnancy should be considered. Drugs with less influence on the fetus are preferred.	Same as the sixth edition	Yes

It could be seen that the guidelines for the diagnosis and treatment of COVID-19 (trial 7^th^ edition) recommended certain treatment principles for severe and critical patients on the basis of symptomatic treatment, active prevention, and treatment of complications of basic diseases, prevention of co-infection, and timely organ function support. Treatment options comprise respiratory support, circulatory support, renal replacement therapy, CP therapy, blood purification therapy, immunotherapy, and others. The authors searched PubMed and found that there have been few studies or reports on the medication analysis of CP therapy coupled with medication therapy for severe COVID-19 patients until now. Therefore, we analyzed the treatment process of patients based on the guidelines newly established in China.

### Antivirals

The diagnosis and treatment of COVID-19 guidelines (trial 7^th^ edition) indicate that antiviral drugs may be tried, including IFN, lopinavir/ritonavir, ribavirin, chloroquine phosphate, and arbidol. In this paper, the antivirals administered to the severe COVID-19 patient contained α-2b IFN, lopinavir/ritonavir, and arbidol.

Lopinavir/ritonavir would elicit a significant anti-coronavirus effect in the early stage, which can reduce the death rate and dosage of glucocorticoids (Jiang et al., 2020). However, if the early treatment window is missed, the efficacy may be reduced. A randomized, open, controlled trial (ChiCTR2000029308) assessed the efficacy and safety of lopinavir/ritonavir in the treatment of COVID-19 patients. The study showed no significant difference between the lopinavir/ritonavir group and the standard-care group in the duration of clinical improvement and mortality (Cao et al., 2020). However, this study did not distinguish whether the antiviral treatment started within 48 hours or 3–5 days or >5 days, which may affect the efficacy analysis. According to the current research results, early use of antiviral drugs such as lopinavir/ritonavir may benefit patients.

By binding to the IFN receptor on the surface of sensitive cells, IFN activates the “antiviral protein” gene and produces antiviral proteins that promote viral mRNA degradation and prevent viral mRNA transcription and translation, inhibiting the replication of the virus. The antiviral effect of IFN may be more substantial in uninfected cells, and patients may benefit more from early use. A SARS study suggested that IFN may exacerbate diseases such as lung damage and even acute respiratory distress syndrome (ARDS) *via* affecting inflammatory factors (Huang et al., 2005). IFN may increase risk in critical patients with multiple organ failure, severe complications, septic shock, and other manifestations, so close pharmaceutical care should be implemented. For the treatment of SARS and MERS, the effective rate and the improvement of clinical symptoms in 14 days with IFN were higher than in the control group, but the effective rate of 28-day treatment was not significant (Chen et al., 2020).

Arbidol has been found to exert an *in vitro* inhibiting effect on coronavirus. This drug has only been approved for sale in China and Russia, and there have been few studies on its efficacy and safety. In patients over 65 years old treated with arbidol (provided that it was administered in the first 48 hours after disease onset), the duration of fever and frequency of complications proved to be lower than in patients who did not receive antiviral therapy in a study reported by Russian (Bulgakova et al., 2017). Reports in the literature and clinical data showed that arbidol was safe for elderly patients.

Lopinavir/ritonavir is mainly metabolized in the liver, and the range of the average plasma protein binding rate is 89.2%-91.6%, which can compete with other drugs in terms of binding to plasma proteins, leading to abnormally high concentrations and adverse drug reactions. Potential drug interactions between lopinavir/ritonavir and CYP3A4 metabolized drugs may also occur; these are summarized in **Table 2**.

**Table 2:** List of potential drug interactions and adverse reactions (Wang et al., 2020).

Drugs	Combined drugs	Risk
**Lopinavir/ritonavir**	HMG-CoA reductase inhibitors	Combination is not recommended: simvastatin. Atorvastatin is recommended under careful monitoring.
**8.1.1**	Sedative-hypnotics	Combination is not recommended: midazolam, triazolam.
**8.1.2**	Extracts of St John's wort (Hypericum perforatum L.)	Combination is not recommended. Combined usage may reduce efficacy.
**8.1.3**	Dihydropyridine Calcium Channel Blockers:	Careful combination. It may lead to an increase in plasma concentration of dihydropyridine calcium channel blockers
**8.1.4**	Oral anticoagulants	During combined use with warfarin, it is recommended to monitor the international normalized ratio (INR).
**8.1.5**	Amiodarone	Careful combination. It may increase the plasma concentration of amiodarone, and the heart rate should be monitored.
**8.1.6**	Triazoles	Combination is not recommended: voriconazole and high-dose itraconazole (>200 mg/d)
**8.1.7**	Immunosuppressants	Careful combination. It may increase the plasma concentration of the immunosuppressant. It is recommended to monitor the concentration of immunosuppressant.
**8.1.8**	Antiepileptic drugs	Combination is not recommended: sodium valproate, lamotrigine. It may be better to adjust the drug dosage according to the blood concentration.
**Ribavirin**	Lamivudine	Careful combination. May cause fatal or non-fatal lactic acidosis.
**8.1.9**	Zidovudine	Careful combination. May reduce the effect of zidovudine.
**8.1.10**	Didanosine	May cause lactic acidosis, liver injury, peripheral neuropathy, and pancreatitis.
**Interferons**	Theophylline	Clearance of theophylline may be reduced. It is recommended to monitor the plasma concentration of theophylline and adjust the dosage of theophylline properly during combined use.
****	Hepatotoxic drugs	Combination with antiepileptics, erythromycin, minocycline, and other hepatotoxic drugs may increase potential risk of liver injury.
**Chloroquine phosphate**	Heparin	Careful combination. During combined use with heparin, it is recommended to monitor the INR.

IFN aerosol inhalation is mentioned in the guidelines (National Health Commission of the People's Republic of China, 2020). The thermal stability of α-2b IFN injection is not good. The use of a jet atomizer (air compression atomizer) or vibrating screen atomizer for atomization is recommended. With oxygen-driven atomization at an oxygen flow rate of 6-8 L/min, 2 hours after atomized inhalation, IFN was distributed in lung tissue. The duration of the concentration of IFN in lung tissue remaining high was about 12 hours, and the clearance half-life was 8–12 hours (Shen et al., 2018). Meanwhile, IFN aerosol inhalation may stimulate the nose, pharynx, and gastrointestinal tract, and adverse reactions of arbidol include nausea, diarrhea, dizziness, and increased serum transaminase. Thus, pharmacists are recommended to watch closely for these symptoms.

The patient in this study was treated with a combination of lopinavir/ritonavir, arbidol, and IFN. Arbidol is a non-nucleoside broad-spectrum antiviral drug that acts by inhibiting the fusion of the virus with the cell membrane. Lopinavir/ritonavir is a protease inhibitor that acts as a treatment for COVID-19 by inhibiting the coronavirus endopeptidase C30 (Shen et al., 2020). IFN was administered by atomized inhalation. The drug was combined with receptors on the surface of respiratory epithelial cells and alveolar cells, inducing the production of various antiviral proteins to establish an “antiviral state” and limiting the further replication and spread of the virus. The three drugs have different mechanisms of action and may have synergistic effects. The adverse reactions to IFN nebulization inhalation are mainly respiratory tract stimulation, a common adverse reaction to lopinavir/ritonavir is diarrhea, and the adverse reactions to arbidol are mainly nausea and diarrhea. The patient developed diarrhea during treatment, which was thought to be related to lopinavir/ritonavir and arbidol. There were no other additional serious adverse reactions. However, clinical studies of three-drug combinations are rare, and the risks and benefits for COVID-19 patients still need to be confirmed by high-quality clinical studies. There are even fewer studies on the safety of a combination of three drugs. In view of the combined drug regimen, it is suggested to strengthen the pharmaceutical care for adverse drug reactions and drug interactions.

Chloroquine has been used for a very long time to treat malaria and showed extraordinary results for virus washout in recent research (Wang et al., 2020). Preliminary clinical studies have found that chloroquine possesses certain inhibitory effects on COVID-19 (Chinese Government Website, 2020). In addition to its antiviral effect, chloroquine has an immunomodulatory effect that can synergistically enhance the antiviral effect. The EC_50_ (half-maximal effective concentration) of chloroquine to SARS-CoV-2 was found to be 1.13 μmol/L, EC_90_ to be 6.9 μmol/L, CC_50_ (half cytotoxic concentration) to be more than 100 μmol/L, and the selection index to be more than 88 (Wang et al., 2020). These indicated good inhibition and low toxicity. In a multi-center clinical trial (Gao et al., 2020), more than 100 COVID-19 patients were included, and the results showed that chloroquine phosphate was superior to the control group in inhibiting the progression of pneumonia, improving the imaging performance of the lungs and promoting the rehabilitation of the disease, and there were few serious adverse reactions when chloroquine phosphate was used. As of May 14, 10 clinical trials of hydroxychloroquine for the treatment of COVID-19 have been registered in the “Chinese Clinical Trial Registry” (three were withdrawn due to there being no new patients). The experimental results of the People's Hospital of Wuhan University showed that hydroxychloroquine can effectively relieve symptoms, reverse the rate of severe disease, and shorten the course of disease (Compilation of information on COVID-19 treatment regimens, ).

The trial sixth edition of the COVID-19 diagnosis and treatment guidelines first mentioned chloroquine phosphate for the treatment of COVID-19 patients. Chloroquine phosphate was recommended for adults aged 18–65 by the NHC. Hydroxychloroquine is an analog of chloroquine, and there have been clinical studies on hydroxychloroquine in the treatment of COVID-19. Both chloroquine phosphate and hydroxychloroquine sulfate contain chloroquine base (chloroquine base is the active ingredient of chloroquine phosphate and hydroxychloroquine sulfate) (Micromedex, 2020). Hydroxychloroquine may cause a variety of clinical adverse reactions, such as the skin system, central nervous system, and cardiovascular system. A few patients exhibit rare symptoms such as hearing impairment, hypoglycemia, and urinary incontinence (Ma et al., 2018).

Epidemiological study showed that about 43.43% of 99 patients with COVID-19 have different degrees of liver dysfunction. In addition to damaging the respiratory system, the virus may also damage other tissue cells such as liver cells (Chen et al., 2020). Lan et al. found that 59.7% of bile duct cells could express SARS-CoV-2 receptor ACE2 while only 2.6% of liver cells could express it, suggesting that the abnormal liver function of patients with COVID-19 may be due to not hepatocyte damage but to cholangiocyte dysfunction and other causes such as drug-induced and systemic inflammatory response-induced liver injury (Chai et al., 2020). In this paper, the patient had no history of liver disease and had mild abnormal liver function (on February 21, aspartate aminotransferase 51.0U/L, γ-glutamyl transpeptidase 162.0U/L, and total bilirubin 28.5µmol/L), which may be related to infection, drug-induced liver injury, and other factors. Combined with the above analysis and the patient's liver dysfunction, the chloroquine regimen was abandoned. We suggest that during the treatment of patients with COVID-19, regular monitoring of liver function should be carried out. At the same time, we suggest that the patients should avoid staying up late, drinking, and other behaviors that could damage the liver after discharge and that regular checks for liver function are essential.

The guidelines issued by the World Health Organization pointed out that there was no evidence to support an effective antiviral treatment for COVID-19, and many potential drug trials are underway. The guidelines recommend symptomatic treatment for mild cases, such as antipyretic drugs, and concern for complications. Oxygen therapy, monitoring of vital signs, and prevention of complications should be employed for severe and critical patients (World Health Organization, ). Based on the current limited clinical data, there is uncertainty in the use of antiviral drugs for the treatment of COVID-19. Therefore, clinical medication monitoring, evaluation, and early warning should be carried out during the treatment, and efficacy and safety evaluation should be conducted regularly. Pharmacists should carry out pharmaceutical care according to the characteristics of the drug and the individual situation of patients to ensure the safety of patients.

### CP Therapy

The CP donors had recovered from SARS-CoV-2 infection and were invited to donate after written informed consent had been obtained. The donors had been previously confirmed COVID-19 and subsequently tested negative for SARS-CoV-2 and other respiratory viruses, as well as for hepatitis B virus (HBV), hepatitis C virus (HCV), HIV, and syphilis at the time of blood donation. The donors had been well (asymptomatic) for at least 10 days, with a serum SARS-CoV-2-specific ELISA antibody titer higher than 1:1000 and a neutralizing antibody titer greater than 40. On February 22 and 24, the recipient received 200 mL and 100 mL of CP, respectively; no adverse reactions such as fever, rash, or dyspnea occurred. After 7 days, the noninvasive ventilator was suspended, the nucleic acid test was negative, and the chest CT showed obvious improvement. No safety risks occurred during the patient's CP treatment.

Plasma therapy is a treatment that involves extracting convalescent blood products, CP, or immune globulin from a cured patient. The CP contains a variety of components of innate immunity and acquired immunity that can effectively fight against pathogenic microorganisms and help recovery from infectious diseases.

During the outbreak of SARS and MERS, plasma therapy reduced the death rate among critical patients, and the earlier the treatment better (Chen et al., 2020). Cheng et al. found that the mortality rate of 80 SARS patients treated with CP (12.5%) was lower than that in relevant areas (17%) during the epidemic period (Cheng et al., 2005). Mair JJ et al. analyzed 32 studies on CP treatment in patients with SARS and severe influenza, and the results showed that CP can significantly reduce the case fatality rate and that early application was more effective (Mair-Jenkins et al., 2015). A meta-analysis showed that CP transfusion within 4 days after the onset of symptoms in patients with Spanish influenza significantly reduced mortality (Luke et al., 2006).

In another report, the inflammatory index and pulmonary imaging examination of 10 severe patients showed statistically significant improvement within 24-48 hours after the infusion of CP. The nucleic acid test of all patients turned negative within 7 days after treatment, and few obvious adverse reactions were observed (Peoples Network, ). In the absence of specific treatment methods, CP is one of the limited means of treatment for critical and severe patients, and not all CP treatments have good results. Arabi et al. found that in 11 patients with MERS-CoV infection, only one person had a high antibody titer (Arabi et al., 2016). The titer of specific protective antibody is an important factor affecting the therapeutic effect (Beigel et al., 2017). In addition, the safety of CP therapy should also be paid attention to. The components of blood are relatively complex and diverse, and adverse reactions caused by blood transfusion occur from time to time. A study on adverse reactions to blood transfusion showed that the main adverse reactions of blood transfusion were allergic reactions, such as fever, sweating, and urticaria (Li et al., 2019). This may be due to the following. (1) There is an allotype of plasma protein in patients with an allergic constitution, and patients with blood transfusion history may produce anti-IgA antibody. (2) Immunoglobulin E in plasma can mediate anaphylaxis (Zhang and Wang, 2014). CP treatment also has potential safety risks. (1) Infection risk: Medical personnel may be infected by exposure to respiratory pathogens when they handle CP (Katz and Tobian, 2014). (2) Safety: Transfusions may lead to infection with other viruses, such as HIV, HBV, and HCV. Plasma infusion may cause anaphylactic shock, transfusion-associated circulatory overload, and transfusion-related acute lung injury (TRALI) (MacLennan and Barbara, 2006). Mora R reported that a patient infected with Ebola developed non-HLA antibody-mediated TRALI after CP therapy (Marta et al., 2015).

### Glucocorticoids and Other Treatments

The use of glucocorticoids in the treatment of COVID-19 is controversial. According to Zhong's study, 18.6% of 1099 patients received glucocorticoid treatment. Among them, the proportion of severe patients was 44.5%, and the proportion of non-severe patients was 13.7% (Guan et al., 2020). However, a paper entitled “Clinical evidence does not support corticosteroid treatment for 2019-nCoV lung injury” issued by the Lancet commented that based on the available evidence, the clinical benefits of glucocorticoids in viral pneumonia are controversial (Russell et al., 2020). The diagnosis and treatment of COVID-19 guidelines (trial 7^th^ edition) recommended short-term treatment with methylprednisolone for patients with disease progression or overactivation of the body or inflammatory response. However, dosage with glucocorticoids is a double-edged sword. Although glucocorticoid can reduce the inflammatory response, it can also inhibit the immune function, especially at high doses and with long-term consumption, which may delay the immune system's clearance of the virus. Hence, we suggested that the doses of glucocorticoids should not exceed 1–2 mg/kg/day equivalent to methylprednisolone, and the patients' blood glucose, blood pressure, and other adverse reactions should be monitored. In this paper, the dose and treatment course of methylprednisone met the guidelines. Routine use of antibiotics is not recommended in guidelines, so the patient was given active treatment of the primary disease and symptomatic support treatment, and the conditions gradually improved. During the treatment, no antibiotics were used, and no bacterial infection occurred.

### Pharmaceutical Care

At present, most versions of diagnosis and treatment guidelines mention antiviral drugs, but most clinicians and pharmacists are not familiar with these. In the absence of specific drugs, there are many options for treatment. Chen reported that 76% of the patients studied received antiviral therapy, including oseltamivir, ganciclovir, and lopinavir/ritonavir, using antibacterial agents such as quinolones, cephalosporins, carbapenems, tegacyclin, and linezolid, and 19% of the patients received methylprednisolone and dexamethasone (Chen et al., 2020). Holshue reported that acetaminophen, ibuprofen, guaifenesin, and remdesivir were used (Holshue et al., 2020). Severe patients usually have a variety of basic diseases as comorbidities, such as cardiovascular diseases and diabetes, which increase the difficulty of treatment, and pharmaceutical care also faces greater challenges.

In view of the above problems, pharmacists should master the combination of drugs, pay attention to adverse reactions to antiviral drugs, such as arbidol, which can easily cause bradycardia, and pay close attention to patients with basic heart diseases. Diarrhea, nausea, and other gastrointestinal reactions were the main adverse reactions reported with short-term administration of lopinavir/ritonavir (Liu et al., 2020). In case of adverse reaction, the drug should be stopped and symptomatic treatment should be provided in a timely manner. Pharmacists are also provided with a list of common risk warnings of potential drug interactions and adverse reactions according to COVID-19 diagnosis and treatment plans combined with the literature (**Table 2**). For example, lopinavir/ritonavir is prohibited to be used in combination with drugs that are highly dependent on CYP3A clearance, such as amiodarone, quetiapine, simvastatin, sildenafil, midazolam, and colchicine. It is also prohibited to be used in combination with CYP3A inducers (rifampin) to avoid lowering the plasma concentration of lopinavir/ritonavir (Bian et al., 2020). In view of the cardiotoxicity of chloroquine phosphate (Luo et al., 2020), patients with cardiovascular diseases such as conduction block, prolonged QT interval, or heart failure should avoid using chloroquine phosphate. Combination of drugs that can cause conduction block and QT interval prolongation with chloroquine phosphate (clarithromycin, itraconazole, moxifloxacin) should also be avoided. The patient was treated with antiviral drugs, insulin, and glucocorticoid. No drug–drug interactions related to lopinavir/ritonavir, IFN, or arbidol were observed, and no adverse reactions or adverse events due to drug–drug interactions occurred during treatment. Lopinavir/ritonavir can be taken with food or after meals. Pharmacists may educate patients to swallow the drug entirety and not chew, break, or crush it. Arbidol and chloroquine phosphate had little effect on the digestive tract. For patients with diabetes, cardiovascular diseases, and respiratory diseases and the elderly, pharmacists should strengthen pharmaceutical care, make up for the lack of clinical attention to multiple drug use, and provide scientific and effective pharmaceutical services for COVID-19 treatment.

The drugs taken by blood donors will also affect the safety of recipients (Shen et al., 2020). One study showed that 11% of the donors did not fully disclose their recent medication history (Melanson et al., 2006). If the drug taken by the donor is teratogenic, the concentration can cause pharmacological effects, or the recipient is allergic to a certain drug in the blood, the recipient will be injured. Therefore, the drugs that need to be monitored include teratogenic and embryotoxic drugs, such as thalidomide, isotretinoin, and warfarin (Schaefer et al., 2006), drugs that affect platelet function, such as NSAIDs (aspirin, clopidogrel, ticlopidine) (Ahrens et al., 2007), 5-hydroxytryptamine reuptake inhibitors (citalopram, paroxetine, sertraline, fluvoxamine, fluoxetine) (Serebruany, 2006), antibiotics that affect platelet function (piperacillin, ampicillin) (He et al., 2001), and genotoxic drugs such as chemotherapy drugs. At present, there is still a lack of clinical data and relevant guidelines on the influence of donors' blood drug types and concentrations on the safety of recipients. In addition to the common adverse reactions to blood transfusion, pharmacists should also be aware of the influence of drugs in the blood of donors on the safety of recipients and the efficacy of the treatment.

## Discussion

At present, frontline clinicians focus on the diagnosis and treatment of COVID-19, while the combination of drugs may be occasionally ignored due to a shortage of medical resources. Exact clinical data on the treatment of COVID-19 with antiviral drugs are still lacking, and their specific efficacy remains to be confirmed by clinical studies. To avoid adverse drug reactions leading to treatment failure, pharmacists should master the latest diagnosis and treatment guidelines, pay attention to the treatment regimen for severe patients and the interaction between basic disease medication and COVID-19 medication, review the doctors' prescriptions in advance, and focus on the usage and dosage, compatibility contraindications, adverse reactions, precautions, and contraindications, providing reasonable drug recommendations for doctors and ensuring the safety of patients.

The COVID-19 epidemic is progressing rapidly, and more and more research is being published online and in academic journals. High-quality studies of CP treatment of COVID-19 are urgently needed. The treatment course of CP, the sequence of antiviral therapy and CP, and the timing of combined application are still inconclusive. Frontline staff need to learn from real and effective treatment and formulate appropriate treatment strategies. Based on the guidelines and clinical practice, we suggest that mild and moderate patients should rest in bed, strengthen supportive treatment, and ensure adequate nutrition. Available antiviral drugs that could be tried are as follows: α-IFN, lopinavir/ritonavir, ribavirin, chloroquine phosphate, and arbidol. CP therapy may be considered for severe cases and in the early stage of critical illness.

## Author Contributions

YW: writing—original draft and conceptualization. YZ: writing—review and editing. QY: validation. KZ: conceptualization and supervision.

## Funding

This research did not receive any specific grant from funding agencies in the public, commercial, or not-for-profit sectors.

## Conflict of Interest

The authors declare that the research was conducted in the absence of any commercial or financial relationships that could be construed as a potential conflict of interest.
